# Quantitative analysis of *Anaplasma marginale* acquisition and transmission by *Dermacentor andersoni* fed *in vitro*

**DOI:** 10.1038/s41598-019-57390-y

**Published:** 2020-01-16

**Authors:** Rubikah Vimonish, Wendell C. Johnson, Michelle R. Mousel, Kelly A. Brayton, Glen A. Scoles, Susan M. Noh, Massaro W. Ueti

**Affiliations:** 1 Program in Vector-borne Diseases, Department of Veterinary Microbiology and Pathology, Washington State University, Pullman, Washington 99164 USA; 2 Animal Diseases Research Unit, USDA-ARS, Pullman, Washington 99164 USA; 3 The Paul G. Allen School for Global Animal Health, Washington State University, Pullman, Washington 99164 USA; 4 Present Address: USDA, ARS, Invasive Insect Biocontrol and Behavior Lab, Beltsville, MD 20705 USA

**Keywords:** Bacteria, Parasitology

## Abstract

In this study, we describe a new *in vitro* tick feeding system that facilitates the study of ticks and tick-borne pathogens. To optimize the system, we used *Dermacentor andersoni* and *Anaplasma marginale* as a tick-pathogen interaction model. Ticks were fed on bovine blood containing 10-fold dilutions of the pathogen to determine the effect of dose on tick infection rate. After feeding on infected blood, ticks were transferred to uninfected blood to stimulate bacterial replication within the tick vector. During stimulation feeding, blood samples were collected daily to determine if infected ticks secreted viable *A. marginale*. The results demonstrated similar attachment rates between the first and second tick feeding. Tick midgut and salivary glands were infected with *A. marginale*. However, salivary gland infection rates decreased as the percentage of parasitized erythrocytes decreased during tick acquisition feeding. Bacteria recovered from the *in vitro* system were able to infect a naïve bovine host. Using the highly transmissible *A. marginale* St. Maries strain, we demonstrated that the artificial tick feeding system is a suitable tool to study tick-pathogen interactions and that *A. marginale* tick salivary gland infection is dose dependent. This work demonstrates the utility of an artificial tick feeding system to directly study the association between the number of acquired pathogens and transmissibility by ticks.

## Introduction

Tick-borne diseases caused by bacteria, protozoa, and viruses are responsible for a significant burden in human beings and animals^1–6^. Recent reports indicate that ticks transmit the majority of emerging arthropod-borne pathogens^7–10^. However, our ability to prevent transmission of tick-borne pathogens is limited. In human beings, the most effective measures rely on tick avoidance. In livestock, prevention of tick-borne pathogen transmission currently depends on the use of acaracides. Unfortunately, acaracides are toxic and, in some regions, losing efficacy^11,12^.

Understanding the key events at the tick-pathogen interface is the foundation for developing strategies to control tick-borne diseases. Currently, such studies require the use of an infected mammal to rear infected ticks^13,14^, thus limiting our ability to tightly control the delivery of the pathogen and accompanying blood meal to the tick vectors. To address this limitation, we have developed a novel *in vitro* tick feeding system. To demonstrate the efficacy of the *in vitro* tick feeding system for controlled pathogen delivery to tick vectors we used *Anaplasma marginale*, which causes bovine anaplasmosis^15^, and one of its natural vectors, *Demarcentor andersoni*^16–18^. *Anaplasma marginale*, an obligate intracellular pathogen in the family Anaplasmataceae, serves as a robust model for acquisition and transmission due to our in-depth understanding of the life cycle of this pathogen within its tick vector^13–15,19^.

In the case of the *A. marginale* transmission model, the male tick takes multiple blood meals and is responsible for transmission, this is called intrastadial transmission because of its occurrence within the adult life stage. In order to complete an infection cycle within the male tick, *A. marginale* must overcome two colonization and replication barriers, first within the midgut and then within the salivary glands^13,20^. During the initial feed, termed the acquisition feed, the pathogen enters and replicates in the tick midgut^13,14,21–23^. When the tick ingests a second blood meal, termed transmission feed, the bacteria transit to and replicate in the salivary glands^13,14,21–23^. The bacteria are subsequently released into the new host with the tick saliva during the transmission feed.

In this study, using the *in vitro* tick feeding system, we first determined if *A. marginale* could successfully complete its life cycle within *D. andersoni* by demonstrating tick midgut and salivary gland infection and the secretion of viable organisms from the tick salivary glands during the transmission feed. Secondly, four *A. marginale* doses were delivered concurrently to four different groups of ticks in order to determine the effect of dose on tick infection rates and the number of bacteria in tick midgut and salivary glands.

## Results

### Tick attachment

**Figure 1:**
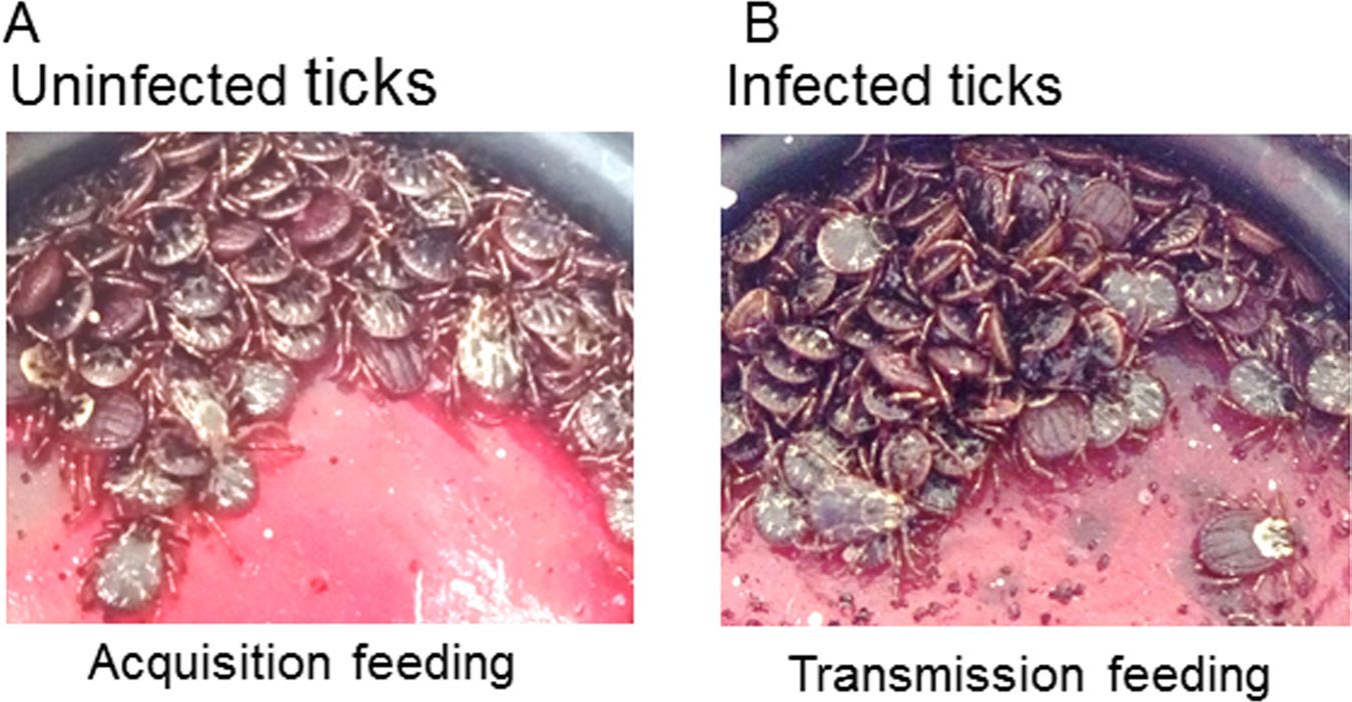
*Dermacentor andersoni* feeding on silicone membrane. (**A**) Uninfected ticks feeding on *A. marginale* infected bovine erythrocytes for acquisition and (**B**) Ticks infected with *A. marginale* feeding on uninfected blood for transmission.

For acquisition feeding, separate feeders containing up to 120 adult male *D. andersoni* ticks were exposed to 10-fold differences in the percentage of parasitized erythrocytes (PPE) from 10^6^ to 10^9^ *A. marginale* per ml of blood (Table 1). The tick attachment rates ranged from 71% to 84% (Table 2). For transmission feeding, 40 to 47 adult male ticks from each group and 10 uninfected female ticks per group were used. The attachment rates for the second feeding ranged from 92% to 96% (Table 2). There were no differences in the tick attachment rates between the four treatment groups during acquisition or transmission feeding (p > 0.35). Figure 1 illustrates ticks attached to the silicone membrane during acquisition (Fig. 1A) or transmission feeding (Fig. 1B).

**Table 1:** *Dermacentor andersoni* feeding on bovine blood infected with *A. marginale*.

Group	Tick exposure	
	*PPE	No. bacteria/ml of blood
1	7.8%	10^9^
2	0.78%	10^8^
3	0.078%	10^7^
4	0.0078%	10^6^

^*^Percentage parasitized erythrocytes determined by Giemsa stained blood smears and number of bacteria by *Msp5*-quantitative PCR.

**Table 2:** Attachment rate of *in vitro* fed adult ticks.

Group	Tick attachment rate	
	Acquisition^a^	Transmission^b^
1	71% (85/120)	92% (53/56)
2	84% (101/120)	96% (48/50)
3	78% (93/120)	92% (52/56)
4	80% (96/120)	94% (54/57)

^a^Ticks fed first *A. marginale* infected blood and ^b^then on uninfected blood.

### Tick acquisition of *A. marginale*

Ticks acquired *A. marginale* from the *in vitro* tick feeding system. Tick midgut infection rates ranged from 80% to 100% (Table 3), with no differences among the four treatment groups (p > 0.76). Tick salivary gland infection rates were 72% in group 1 that received 10^9^ *A. marginale*/ml, 4% in group 2, that received 10^8^ *A. marginale*/ml of blood, and 0% in ticks exposed to lower doses, groups 3 and 4 (Table 3). The infection rate in group 1 was different from the other groups (p < 0.01).

**Table 3:** *A. marginale* infection rates and numbers in *D. andersoni* after acquisition feeding.

Group	Acquisition feeding			
	Midgut		Salivary glands	
	Infection rate^a^	No. *A. marginale*^b^	Infection rate	No. *A. marginale*
1	92% (23/25)	10^6.22 (±0.093)^	72% (18/25)	10^4.45 (±0.107)^
2	100% (25/25)	10^4.87 (±0.090)^	4% (1/25)	10^3.74^
3	80% (20/25)	10^5.00 (±0.101)^	0% (0/25)	—
4	80% (20/25)	10^3.94 (±0.101)^	0% (0/25)	—

^a^Infection rate is the number of positive organs divided by the number tested multiplied by 100. ^b^The number of *A. marginale* is reported as the mean of the log10 transformed data +/− standard error.

Overall, the average number of *A. marginale* in midguts after acquisition feeding, as detected by qPCR, reflected the number of *A. marginale* in the blood meal, and demonstrated that *D. andersoni* ticks were exposed during tick feeding (Table 3). The number of *A. marginale* per midgut in group 1 was 10^6.22 (±0.093)^ bacteria, which was higher than the other 3 groups (p < 0.01). The number of *A. marginale* per midgut was similar in group 2 with 10^4.87 (±0.090)^ and group 3 with 10^5.00 (±0.101)^ receiving 10^8^ and 10^7^ A. *marginale*/ml of blood, respectively (P > 0.99). Group 4, receiving 10^6^ *A. marginale*/ml of blood, had the lowest number of bacteria per midgut at 10^3.94 (±0.101)^ which was different than groups 2 and 3 (p < 0.01).

As expected, the average number of *A. marginale* per salivary gland pair was, overall, comparatively low. Specifically, in group 1 there were 10^4.45 (±0.107)^ bacteria per salivary gland pair (Table 3). In group 2, a single tick was infected with 10^3.74^ bacteria per salivary gland pair, while no *A. marginale* was detected in groups 3 and 4. Due to the low number of infected salivary glands after acquisition feeding, statistical comparison of infected ticks could not be conducted.

### Tick transmission of *A. marginale*

Transmission feeding had little impact on midgut infection rates that ranged from 88% to 96% (Table 4), with no differences among the four groups (p > 0.99). The increased number of *A. marginale* in the midguts after transmission feeding as compared to the acquisition feeding indicated replication, which was particularly evident in group 4 ticks (Table 4), which had approximately a 10 fold increase in *A. marginale* numbers. The number of *A. marginale* per midgut in group 1 was 10 ^6.27 (±0.095)^, which was greater (p < 0.01) than in groups 2 with 10^5.37 (±0.091)^, group 3 with 10^5.20 (±0.091)^ and group 4 with 10^5.00 (±0.092)^ *A. marginale* per midgut.

**Table 4:** *A. marginale* infection rates and numbers in *D. andersoni* after transmission feeding.

Group	Transmission feeding			
	Midgut		Salivary glands	
	Infection rate^a^	No. *A. marginale*^b^	Infection rate^a^	No. *A. marginale*^b^
1	88% (22/25)	10^6.27 (±0.095)^	72% (18/25)	10^5.90 (±0.105)^
2	96% (24/25)	10^5.37 (±0.091)^	60% (15/25)	10^4.30 (±0.114)^
3	96% (24/25)	10^5.20 (±0.091)^	32% (8/25)	10^4.83 (±0.158)^
4	92% (23/25)	10^5.00 (±0.092)^	28% (7/25)	10^4.20 (±0.155)^

^a^Infection rate is the number of positive organs divided by the number tested multiplied by 100. ^b^The number of *A. marginale* is reported as the mean +/− the standard error.

Notably, infection rates in salivary gland pairs were higher in transmission-fed as compared to acquisition-fed ticks (Table 4). The salivary gland infection rate of transmission-fed ticks varied based on the number of *A. marginale* in the blood meal. Specifically, group 1 and group 2 ticks had a 72% and 60% infection rate, respectively. While groups 3 and 4 ticks had infection rates of 32% and 28%, respectively. Group 1 was different from all the other groups (p < 0.01). The number of *A. marginale* per salivary gland pair also increased following the transmission feed, as compared to the acquisition feed, indicating replication of *A. marginale* in the salivary glands (Table 4). For example, the number of *A. marginale* per salivary gland pair increased over 10-fold in group 1 ticks and from non-detectable to >10^4^ in groups 3 and 4. Overall, group 1 ticks had the highest number of *A. marginale* per salivary gland pair at 10^5.90 (±0.105)^, which was different (p < 0.01) from the other three groups and groups 2, 3, and 4 had similar numbers of bacteria per salivary gland pair (p > 0.20) that varied from 10^4.20 (±0.155)^ to 10^4.83 (±0.158)^.

### Pathogen secretion during transmission feeding

Ticks successfully transmitted *A. marginale* into the *in vitro* tick feeding system. In the first four days post attachment on the siliconized membrane, there was no evidence of *A. marginale* secretion into the *in vitro* system. Ticks exposed to higher PPE began secreting quantifiable *A. marginale* into the *in vitro* system on day five. *Msp5*-qPCR demonstrated the number of bacteria secreted into the *in vitro* system at day five and six post tick attachment were 10^4.3^ and 10^3.4^ organisms per ml of blood, respectively.

### Viability of *A. marginale* secreted into the *in vitro* tick feeding system

Intravenous inoculation of 10^5.7^ *A. marginale* from the *in vitro* system infected a naïve, splenectomized animal. Nested *msp*5-PCR detected *A. marginale* in the peripheral blood on day 17 post inoculation. Microscopic examination of Giemsa stained blood smears detected infected erythrocytes on day 29 post inoculation (Table 5).

**Table 5:** Determination of recovered *A. marginale* viability by intravenous inoculation.

Assay	C95064					
	Week post inoculation					
	0	1	2	3	4	5
Nested PCR	N	N	N	P	P	P
Blood smear	N	N	N	N	P	P

Inoculum: 10^5.7^ A. marginale organisms.

Nested PCR positive: day 17 post inoculation.

Blood smear: day 29 post inoculation.

### Stability of *A. marginale* strain throughout the *in vitro* tick feeding system

**Figure 2:**
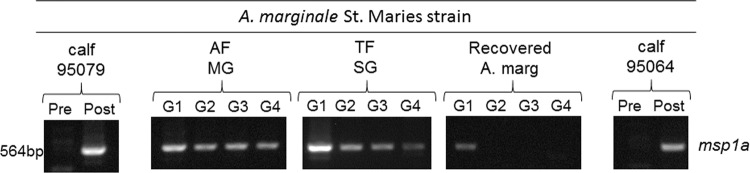
Detection of *A. marginale* St. Maries strain within the vertebrate and invertebrate hosts. Calf 95079: blood donor for the *in vitro* tick feeding system, AF-MG: tick midgut harvested after acquisition feeding, TF-SG: tick salivary gland harvested after transmission feeding, Recovered *A. marginale*: Bacteria secreted into the *in vitro* tick feeding system, and calf 95064: recipient animal inoculated with recovered *A. marginale* from the *in vitro* tick feeding. PCR targeting *msp1a* used for *A. marginale* genotyping. The images were cropped from a single gel image that included molecular weight standard. The full-length gel is presented in Supplementary Fig. 1.

**Figure 3:**
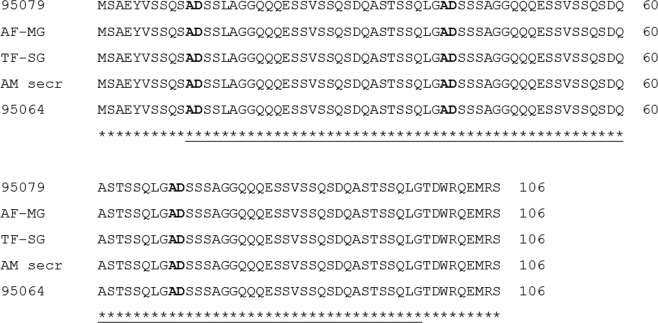
Confirmation of *A. marginale* St. Maries strain identity by sequencing *msp1a* Calf 95079: Blood donor for the *in vitro* tick feeding system, AF-MG: tick midgut harvested after acquisition feeding, TF-SG: tick salivary gland harvested after transmission feeding, AM secr: Bacteria secreted into the *in vitro* tick feeding system, and calf 95064: Recipient animal inoculated with recovered *A. marginale* from the *in vitro* tick feeding. The start of each repeat is in boldface. Solid underlines represent the St. Maries MSP1a repeat region.

*Msp1α* is a single copy gene with tandem repeats that is commonly used for strain typing of *A. marginale*^15,24^. Here we used *msp1α* to confirm the identity and stability of the St. Maries strain throughout the entire infection cycle including the donor calf used for acquisition feeding, midgut following acquisition feeding, salivary glands following transmission feeding, and the recipient calf. The resulting amplicon of 564 base pairs was consistent with the St. Maries strain at all stages (Fig. 2). The images were cropped from a single gel image that included molecular weight standard. The full-length blots/gels are presented in Supplementary Fig. 1. Sequencing confirmed 100% identity among all amplicons at all time points (Fig. 3).

## Discussion

One major limitation in understanding the tick-pathogen interface is the lack of tools to tightly control and alter the delivery of the blood meal to the tick. In the past, various membrane feeding systems used for this purpose lacked consistency in tick attachment and efficiency in acquiring pathogens^25–29^. To address these limitations, we have developed and tested an *in vitro* tick feeding system that can simultaneously deliver at least four different treatments. To test the ability of the system to recapitulate the life cycle of a pathogen within a tick, we used *A. marginale* as a model organism and completed an infection cycle including acquisition and transmission feeds. We concurrently tested the effect of dose on the tick infection rates and number of bacteria in tick midgut and salivary glands.

*In vivo* experiments involving tick feeding on *A. marginale* infected animals are typically conducted either during acute or persistent infection^13,14,19,21,22^. Acute infection is defined as a rising count of infected erythrocytes as detected in Giemsa stained blood smears and >10^8^ *A. marginale* per ml of blood^13,14^. Persistent infection follows acute infection by typically 6 weeks and is defined as microscopically undetectable infected erythrocytes which corresponds to <10^8^ *A. marginale* per ml of blood^21,22^. In our experiments, group 1 and 2 ticks received doses comparable to acute infection (10^9^ and 10^8^ *A. marginale* per ml of blood), while groups 3 and 4 ticks received doses mimicking persistent infection (10^7^ and 10^6^ bacteria per ml of blood).

Because the midgut is the first organ to be infected, midgut infection rates are not expected to differ markedly when comparing the acquisition and transmission fed ticks. *In vivo* infection rates in the Reynold’s Creek colony of *D. andersoni* during acute infection following a seven-day acquisition and transmission feed are typically 100%^13,14,30^. In our experiments, group 1 and group 2 ticks had comparable, but somewhat, lower infection rates between 88 to 100%. The infection rates in the ticks exposed to *A. marginale* mimicking persistent infection in group 3 and group 4 ticks were between 80–92%. The infection rates of *D. andersoni* fed on persistently infected animals with an average of approximately10^6^ infected RBC/ml of blood were between 89–90%^19^. These somewhat lower values do not reduce the utility of the *in vitro* tick feeding system and may be due to some degree of increased variation in tick feeding on the artificial system as compared to cattle. Alternatively, the *A. marginale* doses received by the tick were calculated based on the average number of *A. marginale* /ml of blood for each day of tick feeding. In the artificial feeding system, the number of *A. marginale* are more likely to be more uniform. Thus, the differences in infection may also reflect less variation in the pathogen number in the blood meal.

The number of *A. marginale* in the midguts of the artificially fed ticks is overall comparable to reported values^13,14,19,30,31^. Little published data are available for acquisition fed ticks. In transmission fed ticks exposed to 10^8^ *A. marginale*, the number of bacteria in midgut was approximately 10^6^ organisms^13,14,30^. In our experiments, the ticks fed with 10^8^ *A. marginale* per ml of blood had number of bacteria in midgut of approximately 10^5^ *A. marginale*.

In the salivary glands, the low infection rates in all groups of ticks, except those fed on 10^9^ *A. marginale* per ml of blood, following the acquisition feed is expected based on the lifecycle of *A. marginale* in the tick vector. Following the transmission feed, the infection rates in the salivary glands in group 1 and group 2 ticks, mimicking acute infection, was lower 72% and 60%, respectively, than the 80 to 100% reported for ticks fed on an acutely infected animal (10^8^ *A. marginale*/ml of blood)^13,31^. However, the number of *A. marginale* are overall comparable^13,14,30^. The number of *A. marginale* in the salivary glands following feeding on an acutely infected animal (10^8^ *A. marginale*/ml of blood) was approximately 10^7^ *A. marginale* per salivary gland pair^13^. The ticks receiving a similar dose via artificial feeding had approximately 10^6^ bacteria per salivary gland pair. In ticks fed on a persistently infected animal with 10^6^ *A. marginale* per ml of blood, the number of pathogens in the salivary glands were 10^4^ organisms per salivary gland pair^19,32^, similar to the group 4 ticks, which also had 10^4^ *A. marginale* per salivary gland pair.

The effect of dose on tick infection rates and number of bacteria was only apparent in the salivary glands, indicating the salivary glands are a stronger barrier to pathogen infection as compared to the midgut. This echoes previously published data in which super-infection exclusion only occurred in *D. andersoni* salivary glands, but not midguts when ticks were exposed to two strains of *A. marginale* during sequential tick feeds^33^. This suggests that targeting the interaction between the pathogen and the salivary gland may lead to more effective interventions than targeting the pathogen-midgut interface.

Importantly, *A. marginale* was detected in the blood receptacle following transmission feeding of the tick vector. Additional experiments are required to quantitate the number and viability of *A. marginale* secreted through time using this system. To date, *A. marginale* present in the salivary secretions during transmission feeding has not been quantified. Thus, there is no reference point for the number of *A. marginale* secreted during feeding on the artificial system. The infection of an inoculated calf confirmed viability of the organism and successful completion of an infection cycle. In a previous study, inoculation of multiple calves with residues of erythrocytes collected from artificial membrane systems during *A. marginale*-infected adult ticks feeding failed to infect naïve bovine hosts. Artificially fed adult ticks did acquire and transmit *A. marginale* as demonstrated when exposed ticks infected naïve animals, however, due to the lack of quantitative methods, it was not possible to enumerate the number of *A. marginale* per salivary gland pair dissected from artificially fed ticks^34^. In another study, *D. variabilis* adult ticks fed *A. marginale* blood via a capillary feeding system developed midgut infections. However, following a second feeding on sheep*, A. marginale* did not disseminate to the salivary glands as would be expected^35^.

In conclusion, we have demonstrated that the life cycle of *A. marginale*, including intrastadial transmission, as has been well defined experimentally *in vivo*, is recapitulated via an *in vitro* tick feeding system. Additionally, pathogen dose delivered to the tick has an effect on infection rates and the number of bacteria in tick salivary glands, but not in the midgut. Although *A. marginale* was used as the model organism in this system, it may be adapted for other tick species and pathogens. However, the efficiency of transstadial transmission, epidemiologically relevant for many tick borne pathogens, must be determined^31^. The use of siliconized membranes is not suitable for all studies, particularly those involving the dermal tick-pathogen-host interface. However, the *in vitro* tick feeding system presented here mimics the host micro-environment for tick feeding success. Thus, it may serve as an essential tool to dissect the invasion, colonization, and transmission mechanisms of pathogens and may provide a framework for reduction and refinement of animal use to study ticks and tick-borne pathogens of human and veterinary importance.

## Materials and Methods

### Bovine blood infected with *A. marginale*

For acquisition feeding, blood was collected daily from an acutely infected animal, Calf 95079, and used to infect *D. andersoni* ticks through the *in vitro* tick feeding system. Four *A. marginale* doses were delivered concurrently, each to a different group of ticks (Table 1).

### *In vitro* tick feeding system

**Figure 4:**
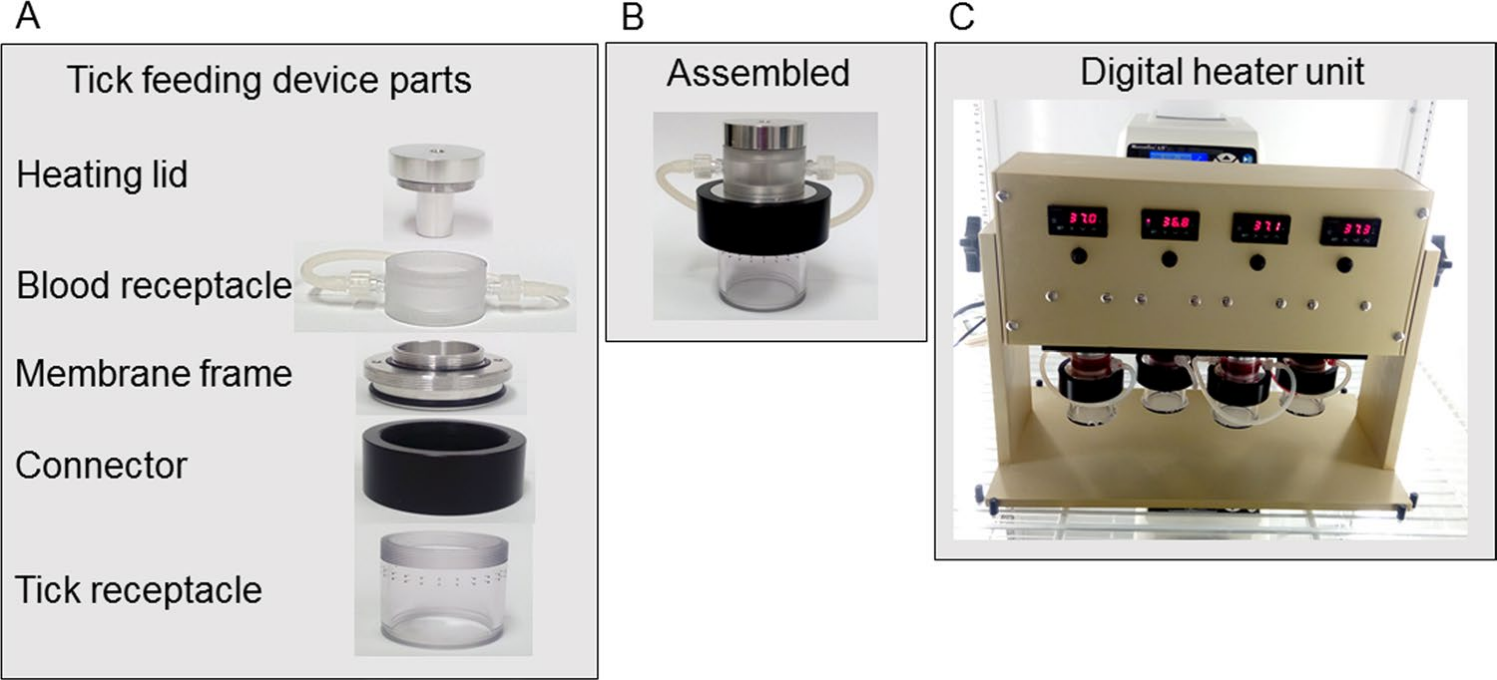
*In vitro* tick feeding system. The unassembled feeder (**A**) is composed of a heating lid, blood receptacle, frame to support the membrane, a connector and a tick vessel. Unassembled feeder; (**B**) Assembled feeder and (**C**) A digital heating power source with four tick feeding device with a peristaltic pump in the back.

The system includes a tick feeding chamber, a digital heating power source and peristaltic pump. The feeding chamber consists of five parts assembled by threads: (1) blood heating element, (2) blood receptacle, (3) tick vessel, (4) connector, and (5) membrane frame (Fig. 4A,B). The simple assembly and disassembly of the feeding chamber allows cleaning and blood changing without interrupting tick feeding. The blood receptacle holds 20 ml of blood. The mammalian host blood temperature is mimicked through a heating element connected to a digital heating power source (Fig. 4C), that can support four independent feeders simultaneously. When blood circulation is necessary, the system can be connected to a peristaltic pump with a velocity of 70 ml per min through siliconized C-Flex Ultra tubing (Cole-Parmer, Vernon Hills, IL). The tubing is connected by two luer lock outlets on the side of the blood receptacle. Each tick feeding vessel holds approximately 100 to 150 adult ticks. Ticks are confined in the vessel by a white silk screen mesh. The entire system is placed in an incubator with a controlled temperature at 26 °C with a 14 hr light/dark photo period. Maintaining the system at a constant 26 °C provides an environment that encourages tick migration and attachment to the membrane covering the blood that is heated to 37 °C.

### Feeding membrane preparation

Siliconized membranes were prepared using commercially available Goldbeater’s skin (Talas, Brooklyn, NY). Five ml of each Ecoflex Supersoft 00–50 silicone components A and B (Smooth-On, Easton, PA) were mixed and thinned with 0.75 ml of hexane (Sigma-Aldrich). Goldbeater’s membranes were taped to a smooth surface and saturated with the silicone mixture to yield an approximately100 μm thick membrane. Membranes were air dried overnight at room temperature.

### Animals, tick vector and pathogen

The animal use in this study (IACUC #2018-16) was approved by the Institutional Animal Care and Use Committee of the University of Idaho, Moscow, Idaho, in accordance with institutional guidelines based on the U.S. National Institutes of Health Guide for the Care and Use of Laboratory Animals. Two five-month-old Holstein calves were confirmed to be free of *A. marginale* as determined by the *Msp5*-ELISA serologic test (VMRD, Pullman, WA) and *Msp5*-nested PCR^14,36^. The specific pathogen-free Reynolds Creek colony of *D. andersoni* and readily transmissible St. Marie’s strain of *A. marginale* were used in this study^13,17,21,22^.

### Tick acquisition feeding

Calf 95079 was inoculated intravenously with blood stabilate containing about 10^9^ bacteria per ml. During acute infection, 100 ml of defibrinated blood was collected daily to feed ticks on the *in vitro* tick feeding system. Microscopic examination of Giemsa stained blood smears and *Msp5*-qPCR were used to assess the number of bacteria^13,17^. Blood was centrifuged to deplete the white blood cells and reconstituted to 30% packed cell volume with 0.2% glucose and stored at 4 °C. Prior to tick feeding in the *in vitro* system, blood was warmed to 37 °C. Uninfected blood was collected in the same manner of infected blood and reconstituted to 30% packed cell volume and 0.2% glucose and used to dilute the infected blood. Four *in vitro* tick feeding devices each containing bovine blood with 10-fold dilutions of *A. marginale* were used to feed the ticks. Each blood meal was changed every 8 hours. Each tick feeding device received 100 male and 20 female *D. andersoni* ticks. Though epidemiologically irrelevant for intrastadial transmission, the female ticks were added to the feeding chamber to stimulate the attachment of male ticks to the silicone membrane. Ticks were allowed to feed on the *in vitro* system for seven days to acquire *A. marginale*.

After acquisition feeding, the ticks were incubated at 26 °C in 94% relative humidity for five days. This interval ensured that the blood meal was completely digested. After incubation, a cohort of 25 male ticks were dissected and midgut and salivary glands harvested. Genomic DNA was extracted to confirm entry into the midgut epithelial cells and if colonization had occurred using *Msp5*-qPCR as previously described^17^.

### Tick transmission feeding

Defibrinated blood was collected daily from an uninfected calf to feed ticks in the *in vitro* tick feeding system. Blood was centrifuged to remove the white blood cells. The blood was reconstituted to 10% packed cell volume, the minimum needed to support tick feeding, with 0.2% glucose and stored at 4 °C. Prior to feeding ticks in the *in vitro* system, blood was warmed to 37 °C. Four *in vitro* tick feeding devices, containing uninfected bovine erythrocytes, were used to feed ticks for transmission. Each feeding device contained 40 to 47 male and 10 uninfected female *D. andersoni* ticks. Ticks were allowed to feed for six days to stimulate *A. marginale* replication in the salivary glands. After transmission feeding in the *in vitro* system, ticks were dissected to determine *A. marginale* replication in the midgut and salivary glands by *Msp5*-qPCR^17^. Blood samples from the *in vitro* system were collected and DNA extracted to determine secretion of *A. marginale* into the *in vitro* tick feeding system.

### Viability and infectivity of *A. marginale* through inoculation of a naïve animal

During the transmission feed, blood samples from the *in vitro* system were collected every 8 hours, DNA extracted and *Msp5*-qPCR performed to determine if *A. marginale* was secreted into the *in vitro* system during tick feeding. To evaluate infectivity, approximately 5 ml of blood recovered from the *in vitro* system during transmission feeding was inoculated intravenously into naïve splenectomized calf 95064. Blood samples from calf 95064 were collected daily and monitored by microscopic examination of Giemsa stained blood smears and *Msp5-*nested PCR^37^.

### DNA extraction

Genomic DNA was extracted from blood by using a PureGene DNA Isolation kit following the manufacture’s guideline of (ThermoFisher Scientific, Waltham, MA). For tick midgut and salivary gland, samples were lysed in 450 μl of Tris-EDTA and sodium dodecyl sulfate (SDS) with 50 μl of Proteinase K (2 mg/ml) and incubated overnight at 55 °C. Following the incubation, 1 μl of glycogen (10 μg/ml) was added to the samples. The proteins were precipitated with ammonium acetate. Genomic DNA samples were precipitated in isopropanol, washed with 70% ethanol, and suspended in 50 μl Tris-EDTA.

### TaqMan quantitative PCR

*Msp5*, a, highly conserved single copy gene in *A. marginale*, was used to quantify bacteria in all samples^15,17^. The primer sequences (forward 5’-CTTCCGAAGTTGTAAGTGAGGGCA-3′; reverse 5′-CTTATCGGCATGGTCGCCTAGTTT-3′) amplify a 202 bp fragment and a TaqMan probe (5′-GCCTCCGCGTCTTTCAACAATTTGGT-3′) binds between the primer sets as previously described^13,17^. Reactions were performed using SsoAdvanced Supermix, 50 µM each primer, 100 µM TaqMan probe, and 5 µl of template DNA. Thermocycling conditions consisted of 45 cycles of 95 °C for 3 min, melting at 95 °C for 15 sec, and annealing at 55 °C for 45 sec, with extension at 72 °C for 7 min. The assay was performed using a Biorad CFX real-time PCR detection system (Bio-Rad Laboratories, Hercules, CA). Standard curves were constructed by amplification of a serially diluted plasmid standard from 10^6^ to 10^2^ *msp5* copies, as previously described^13,17^. Quantitative PCR was conducted in triplicate for each sample.

### Nested PCR

*Msp5* was used as the gene target for nested PCR. External primer sets of forward (5′-GCATAGCCTCCGCGTCTTTC-3′) and reverse (5′-ACACGAAACTGTACCACTGCC-3′) were used to amplify a fragment of 525 base pairs^13,17^. Reactions containing 2 μl of template DNA, 1 μl of 10 µM of each primer, 10 µl of RedTaq (Sigma-Aldrich) and 6 µl of nuclease free water (Ambion) under the following conditions: one cycle at 95 °C for 4.5 min, 35 cycles of 95 °C for 30 sec, 65 °C for 1 min, 72 °C for 1 min, and extension at 72 °C for 5 min. Internal primer sets of forward (5′- TACACGTGCCCTACCGAGTTA-3′) and reverse (5′- TCCTCGCCTTGGCCCTCAGA-3′) were used to amplify a fragment of 343 base pairs^13,17^. Nested reactions were performed using 0.5 μl of external PCR product, 1 μl of 10 µM of each primer, and 10 µl of RedTaq (Sigma-Aldrich) under the following conditions: One cycle at 95 °C for 4.5 min, 35 cycles of 95 °C for 30 sec, 55 °C for 30 sec, 72 °C for 30 sec, and extension at 72 °C for 5 min. Following electrophoresis, amplicons were visualized on a 2% agarose gel.

### Strain-specific PCR

Primer sets of forward (5′-GTGCTTATGGCAGACATTTCC-3′) and reverse (5′-CTCAACACTCGCAACCTTGG-3′) were used to amplify the 5′ end of *Msp1a*, which has tandem repeats used as strain markers as previously described^1,14,24^. PCR was performed using 1 μl of template DNA, 1 μl of 10 µM of each primer, and RedTaq (Sigma-Aldrich) under the following conditions: One cycle at 95 °C for 3 min, 35 cycles of 95 °C for 30 sec, 55 °C for 30 sec, 72 °C for 45 sec, and extension at 72 °C for 7 min. Amplicons were visualized by 1% agarose gel electrophoresis and PCR products sequenced (Eurofins Genomics, Louisville, KY).

### Statistical analysis

An analysis of variance was conducted for *A. marginale* numbers, tick attachment, and infection rate. A mixed model was used with binomial tick attachment data where ticks were considered positive if *A. marginale* was detected with *Msp5*-qPCR and fixed effects were type of tick feeding and *A. marginale* dose and including a repeated effect of individual tick (SAS 9.4 GLIMMIX, SAS^®^ Inst. Inc., Cary, NC). The mixed model for comparisons of binomial infection rate (GLIMMIX) and continuous and greater than 0 *A. marginale* (MIXED) included fixed effects of tissue sample, *A. marginale* dose, and type of tick feeding with a repeated effect of individual tick. *A. marginale* per salivary gland pair was transformed to log10 due to heterogeneous variances between groups. Pairwise multiple comparisons were conducted with Tukey’s test.

## Supplementary information

Suplementary information.

## Data Availability

All data generated or analyzed during this study are included in this published article.
