# Supplementary material for: Quantitative analysis of Anaplasma marginale acquisition and transmission by Dermacentor andersoni fed in vitro
Source: Sci Rep. 2020 Jan 16;10:470. doi: 10.1038/s41598-019-57390-y (PMC6965182; doi:10.1038/s41598-019-57390-y)

Quantitative analysis of *Anaplasma marginale* acquisition and transmission by *Dermacentor andersoni* fed *in vitro*

Rubikah Vimonish^1^, Wendell C. Johnson^2^, Michelle R. Mousel^2,3^, Kelly A. Brayton^1^, Glen A. Scoles^2,#^, Susan M. Noh^1,2,3*^, Massaro W. Ueti^1,2,3,*^

^1^Program in Vector-borne Diseases, Department of Veterinary Microbiology and Pathology, Washington State University, Pullman, Washington 99164, USA.

^2^Animal Diseases Research Unit, USDA-ARS, Pullman, Washington 99164, USA.

^3^The Paul G. Allen School for Global Animal Health, Washington State University, Pullman, Washington 99164, USA.

^#^ Current address: USDA, ARS, Invasive Insect Biocontrol and Behavior Lab, Beltsville, MD 20705

Supplementary Figure 1


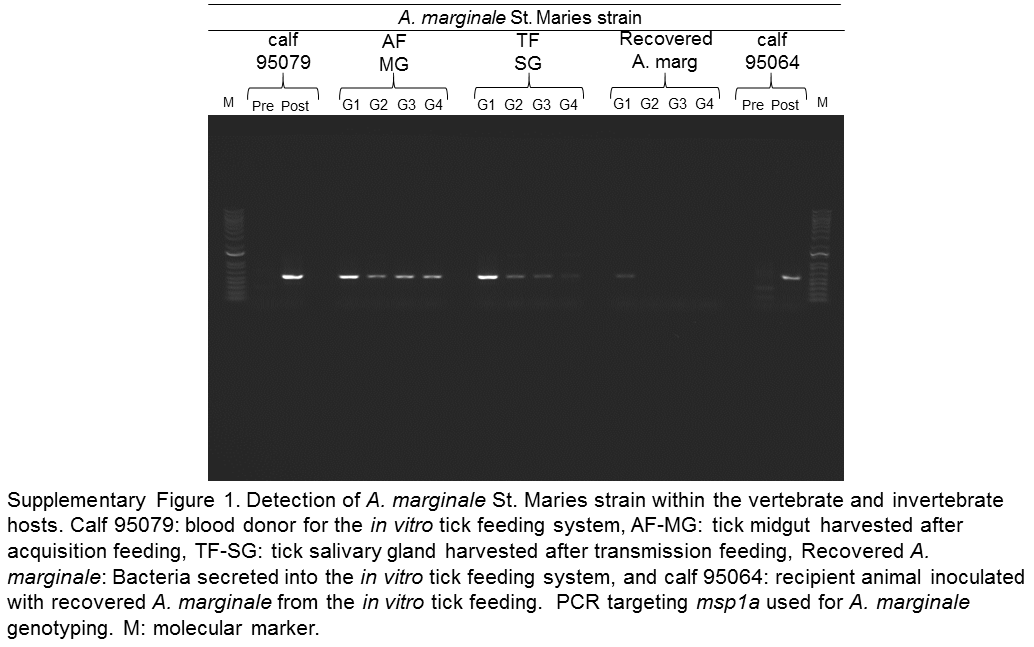

Supplement: Supplementary file 1 — Suplementary information. [file 41598_2019_57390_MOESM1_ESM.docx]
